# Children's emotional experience two years after an earthquake: An exploration of knowledge of earthquakes and associated emotions

**DOI:** 10.1371/journal.pone.0189633

**Published:** 2017-12-20

**Authors:** Daniela Raccanello, Roberto Burro, Rob Hall

**Affiliations:** 1 Department of Human Sciences, University of Verona, Verona, Italy; 2 Environmetrics Ltd, Pymble, Australia; University of L'Aquila, ITALY

## Abstract

A major earthquake has a potentially highly traumatic impact on children’s psychological functioning. However, while many studies on children describe negative consequences in terms of mental health and psychiatric disorders, little is known regarding how the developmental processes of emotions can be affected following exposure to disasters.

**Objectives:**

We explored whether and how the exposure to a natural disaster such as the 2012 Emilia Romagna earthquake affected the development of children’s emotional competence in terms of understanding, regulating, and expressing emotions, after two years, when compared with a control group not exposed to the earthquake. We also examined the role of class level and gender.

**Method:**

The sample included two groups of children (*n* = 127) attending primary school: The experimental group (*n* = 65) experienced the 2012 Emilia Romagna earthquake, while the control group (*n* = 62) did not. The data collection took place two years after the earthquake, when children were seven or ten-year-olds. Beyond assessing the children’s understanding of emotions and regulating abilities with standardized instruments, we employed semi-structured interviews to explore their knowledge of earthquakes and associated emotions, and a structured task on the intensity of some target emotions.

**Results:**

We applied Generalized Linear Mixed Models. Exposure to the earthquake did not influence the understanding and regulation of emotions. The understanding of emotions varied according to class level and gender. Knowledge of earthquakes, emotional language, and emotions associated with earthquakes were, respectively, more complex, frequent, and intense for children who had experienced the earthquake, and at increasing ages.

**Conclusions:**

Our data extend the generalizability of theoretical models on children’s psychological functioning following disasters, such as the dose-response model and the organizational-developmental model for child resilience, and provide further knowledge on children’s emotional resources related to natural disasters, as a basis for planning educational prevention programs.

## Introduction

### The 2012 Emilia Romagna earthquake

A major earthquake has a potentially highly traumatic impact on people’s psychological functioning [1–3]. This is notably true for children, who are highly vulnerable to the traumatic consequences of disasters, and whose vulnerability depends on the level of cognitive and emotional development [4]. There is an emerging body of research studies on the psychological impact of disasters focused on children, particularly considering that the previous focus of many studies has been with adults [5,6]. However, while most of these studies on children describe negative consequences in terms of mental health and psychiatric disorders, little is known regarding how the developmental processes of emotions can be affected following exposure to disasters.

This study refers to the 2012 Emilia Romagna earthquake, a seismic event consisting of a series of shocks that took place in the Emilian Po Plain, Northern Italy; mainly in the provinces of Modena and Ferrara [7,8]. Over a period of seismic activity, two particularly intense shocks were registered in May. The first, a 5.9-magnitude earthquake, was registered on May 20, 2012, at 04:03 local time. The epicenter was 17 km away from Mirandola, a town in the province of Modena, and the fault rupture was estimated at being between 6.3 km and 9 km deep [8]. The second, a 5.8-magnitude earthquake, occurred in the same zone on May 29, 2012, at 09:00 local time. This time the epicenter was 2 km away from Mirandola and fault rupture around 10 km deep [7]. Aftershocks were felt for subsequent months, causing distress for the population in the area [9].

The two earthquakes claimed 27 victims: They were all adults; two-thirds of whom died while working [7,8]. The May 20 earthquake caused the death of seven people, injured about 50 others, and left more than 5,000 people in need of shelter [8]. The May 29 earthquake caused the death of 20 people (three of whom died during the following days), injured at least 350 people, and increased to about 15,000 the number of people who had to leave their homes [7]. Damage to residential buildings was not severe, but the number of people needing alternative accommodation increased during the subsequent weeks, due to people’s reluctance to return home and to the practice of cordoning off town centers. However, the earthquakes caused severe economic damage due to structural damage to industrial buildings and historic structures, and the subsequent closure of industrial facilities [7].

In this work, we focused on whether and how some components of emotional competence–as the ability to understand, regulate, and express emotions [10]–amongst children who were exposed to the 2012 Emilia Romagna earthquake contrasted with those of a control group after two years.

### The impact of natural disasters on children’s psychological functioning

The literature documents a wide spectrum of psychological outcomes ranging from very severe impairment of mental health to increased resilience in both adults and children who experienced a disaster [4,6,9]. Increases in resilience reflecting effective coping and adaptation in the face of major life stress [6]. However, there are some inconsistencies in the data on the prevalence of children’s posttraumatic reactions [7,11]. These reactions commonly include anxiety and depression symptoms related to a range of psychopathologies such as posttraumatic stress disorder (PTSD), panic and anxiety disorders, phobias, and depression [6,7]. While it is not surprising that these effects can occur in the aftermaths of a disaster, we might expect these effects to decrease over the long-term. Unfortunately, this is not always the case [12,13].

The variation in trauma prevalence is probably due to the variety of factors that affect posttraumatic reactions, including the nature of the exposure, age, and gender. These factors–along with others such as resource loss, social support, or parent-child interactions–are conceptualized as risk and protective factors within the dose-response model, one of the theoretical frameworks most used to predict youth’s functioning after traumatic events [6,11,14]. Children’s exposure refers to physical proximity and perceived threat to the self. Meta-analytic studies indicated that exposure is a stronger predictor of children’s posttraumatic symptoms compared, for example, to disaster type, i.e., natural, technological, or human-made [8,13]. For both age and gender, findings are not consistent [8,9,13]. For example, preschool children demonstrated antisocial and aggressive behaviors more frequently compared to older children, but other symptoms such as PTSD differed according to age [8,9,13]. Males frequently displayed externalizing behaviors such as aggression, while females displayed internalizing behaviors such as depression and PTSD. However, confounding biases could be linked to difficulties distinguishing effects of experiencing versus reporting symptoms [5,15,16]. The loss of objects, such as homes or properties, and conditions, such as health, employment, or other personal and social resources, is a strong risk factor for psychological distress and PTSD for adults, and it seems to have similar effects for children [14]. Finally, social support is a relevant protective factor against negative outcomes such as PTSD and promotes positive outcomes such as post-traumatic growth for children [14]. Similarly, parent-child interactions are very salient in post-disaster contexts, with parental trauma reactions markedly affecting offspring’s functioning, especially for younger children [14].

Even if negative emotional reactions such as fear, distress, and anger are quite common in people affected by a disaster [17], exposure could in some cases represent a positive “turning point experience” [18]. Some studies highlight for example that children exposed to disasters such as tornados are resilient in the face of adversity, with recovery depending upon internal or external protective factors like self-regulation or school reestablishment [9]. A few studies have also documented how exposure to a natural disaster, and to an earthquake in particular, impacts children’s cognitive or social domains [3,19,20], but studies on the impact of disasters on the development of emotional competence are lacking.

### Emotional competence and traumatic events in children

Emotional competence could play an important role in relation to children’s responses to exposure to traumatic events, given its contribution to adjusting in social life in general [10]. It might also play a similar role in adaptation to adversity as conceptualized within theoretical models such as the organizational-developmental model for child resilience [21]. Through adaptation, children actively use internal and external resources (e.g., biological, cognitive, emotional, etc.) to master stage-salient tasks. The model also postulates that in early childhood, adaptation would be related mainly to the emotional responsiveness or involvement of parents. Then, during middle childhood internal resources such as socio-emotional competencies would play a major role. However, to our knowledge there are no studies examining how emotional understanding, regulation, and expression are affected long-term in children exposed to natural disasters.

First, understanding emotions is a complex ability comprising sub-components, which gradually develops from preschool to school years during three hierarchical developmental periods, usually in similar ways for males and females [22,23]. At about five, children understand public elements of emotions; at about seven, they understand their mentalistic nature; at about nine, they understand their reflexive connotation [22,23]. However, exposure to traumas such as maltreatment can affect the development of this ability, impairing for example children’s knowledge about sources of affective information [24].

Second, emotional regulation, as “the ability to decrease, maintain or increase one’s emotional arousal to facilitate engagement with the context” (p. 625 [25]), is related to healthy socioemotional development and emergence of psychopathology [26]. It plays a fundamental role in explaining the beginning and maintenance of symptoms such as anxiety and depression, particularly for maltreated children [26]. However, the findings are not consistent with respect to age and gender differences: For example, coping strategies become more refined at increasing ages, but no differences in emotion regulation strategies emerge–either for age or gender–when the informants are significant adults [25,27].

Third, little is known regarding how children express emotions associated with their knowledge of natural disasters and earthquakes in particular, and associated scripts. Nevertheless, prior knowledge concerning risky phenomena is, at least for adults, a factor promoting actions taken to mitigate damage caused by earthquakes [28,29]. At three, children are already able to produce script narratives, as “general descriptions of what usually happens in an event”, related to novel events even after single occurrences (p. 91 [30]). This ability becomes refined with increased age and increased experience: Children gradually include affective and cognitive internal states [30,31], and scripts are more accurate when their learning processes refer to behaviors rather than facts [32]. However, only a few studies have examined how children develop their knowledge of earthquakes [33–35]. A study with kindergartners through six-graders who had experienced different levels of seismic activities indicated that they can report causes and descriptions of earthquakes, but they rarely had adequate scientific knowledge [35]. Again, Turkish first to six-graders with different levels of exposure demonstrated gaps in their scientific knowledge on causes and consequences of earthquakes [34]. More recently, a study with focus groups involving nine and ten-year-olds in New Zealand revealed that they seemed well informed about the causes, characteristics, and consequences of earthquakes [33]. In addition, they reported fear as the most intense emotion associated with earthquakes, but excitement or anger were also mentioned [33]. However, in these studies the role played by exposure, age, or gender was not systematically investigated, nor were emotional language or emotional intensity analyzed.

Children develop the ability to use emotional terms to describe their internal state quite early in life [36]. By the age of two/three, they use verbal labels to describe their own and others’ emotions such as fear, sadness, anger, and enjoyment, and during the preschool years they include these labels in conversations and narratives of past and future personal events of different valence [36,37]. This helps them make sense of personally experienced events that may become part of their autobiographical memory; a process particularly relevant in the case of stressful and traumatic events in which people must search for meaning within apparently senseless events [37]. Factors such as exposure to a stressful event, age, or gender can contribute to the explanation of differences in children’s use of emotional language in recounting memories of negative events. For example, narratives of preschoolers who had experienced more severe damage during the Hurricane Andrew (Florida, US, 1992) had fewer positive emotions a few months after, and a relatively higher number of negative emotions after six years [38]. Some authors have nevertheless suggested that the presence of negative emotional terms could be a manifestation of traumatic symptoms [39]. As age increases, children have also been shown to demonstrate higher introspective abilities expressed in terms of richer emotional language, for example for five to nine-year-olds narrating personally experienced suffering and wellbeing events [40]. However, there have been inconsistent findings, for example comparing short-term and long-term memories on tornados [38,41]. Finally, gender differences were documented in terms of preschool girls’ richer production of emotional mental words in parent-child conversations [42], while differences were not consistently revealed in studies on narratives of personal positive and negative events in primary school children [40,43].

#### Aims

We explored whether and how the exposure to a natural disaster such as the 2012 Emilia Romagna earthquake affected the development of children’s emotional competence in terms of understanding, regulating, and expressing emotions [10], when compared with a control group not exposed to the earthquake. We operationalized the expression of emotions by examining the emotional language which children used when discussing earthquakes and the intensity with which the emotions were characterized. The earthquake took place when children were five and eight-year-olds, and the data were collected when they were seven and ten-year-olds, respectively. We examined also the role of class level and gender, taking into account the complex pattern of effects previously described. For the experimental group, we also checked preliminarily for differences related to the type of damage (to persons or economic), on the bases of findings relating to resource loss [14]. Beyond assessing the children’s understanding of emotions and regulating abilities with standardized instruments [23,44], we employed semi-structured interviews to explore their knowledge of earthquakes and associated emotions, and a structured task on the intensity of some target emotions. To our knowledge, no previous study addressed these issues.

We had three main aims.

(1) The first aim was to investigate whether and how children exposed to the earthquake differed from the control group for their understanding and regulation of emotion, exploring age and gender differences. We expected:
  - exposure to have a negative influence on the understanding and regulation of emotions, based on findings about maltreated children [24,26];
  - understanding (but not regulation) of emotions to increase with age [22,23,25,26];
  - no gender differences for either understanding or regulation [22,23,25,26].
(2) The second aim was to explore whether and how children embedded affective elements into their knowledge of earthquakes. As a preliminary step, we examined the role of earthquake exposure, class level, and gender on children’s explanations of earthquakes, in terms of plausibility, length, and complexity of content spontaneously reported (including affective elements). We formulated the following hypotheses.
  - We expected most of the children to exhibit at least minimal plausible knowledge of earthquakes, even if not refined from a scientific perspective, in light of the cognitive and linguistic abilities already possessed by primary school children, and the development of naïve biological, physical, and psychological theories enabling them to make ontological distinctions and to identify domain-specific causal explanations [45,46], and specific knowledge of earthquakes [33–35].
  - We hypothesized higher length and more differentiated content types for children exposed to the earthquake compared to the control group, and for older compared to younger children, in light of the role of experience for the development of more complex scripts [30,31], of the advantage of basing on behaviors rather than facts for successful formation of scripts [32], and of more general developmental acquisitions [9,45,46].

We had no specific hypothesis about the role of gender due to the absence of previous findings.

In addition, we explored the salience in children’s representations of earthquakes of different content categories related to the material domain, such as natural and man-made aspects, or the individual domain such as biological, behavioral, cognitive, and affective aspects. We also investigated the temporal relations between the content categories and the description of earthquake shocks provided by the children. Concerning the affective elements, we examined whether children embedded them spontaneously into descriptions of an earthquake as an indicator of their relevance, even if they usually have a peripheral role among the scripted elements of an event for children of this age [30,31].

(3) The third aim of this research was to compare the emotional language used spontaneously by children describing their knowledge of earthquakes with that used following emotional prompts (expecting the prompts to trigger more emotional expressions than where used spontaneously). Based on previous data on the development of children’s abilities to properly use the emotional lexicon to connote a personally experienced event [36,37], we expected the children to refer to the emotions associated with an earthquake in ways that were like those reported in the trauma literature [6,7,17]. Specifically, we hypothesized:
  - negative emotions to be more frequent and intense for children exposed to the earthquake compared to the control group, confirming findings on long-term memory of traumatic events such as tornados [38] and completion of stories [13];
  - the emotional language to be richer at increasing ages on the bases of children’s more elaborated cognitive, linguistic, and emotional abilities [45,46];
  - more frequent and intense anger for boys and sadness for females, on the bases of adolescents’ different display of externalizing and internalizing behaviors after disasters [8,15,16];
  - negative emotions to be more frequent and intense than positive emotions, and fear to be more frequent and intense than other emotions [17,33].

## Method

### Participants

The sample included two groups of children (*n* = 127) attending primary school in Northeastern Italy in March 2014, when we collected the data for this study: The experimental group (*n* = 65) experienced the 2012 Emilia Romagna earthquake, while the control group (*n* = 62) did not. The participants met two selection criteria: being physically present in the same town where we gathered the data when the 2012 Emilia Romagna earthquake hit, both on May 20 and 29, and not being certified for mental health disturbances prior to the data gathering. Because of the exploratory nature of the study, we used a convenience sample.

Children in the experimental group had experienced the 2012 Emilia Romagna earthquake and were attending the second grade (*n* = 29; *M*_age_ = 7;7, range = 7;0–8;2; 62% females) or the fifth grade (*n* = 36; *M*_age_ = 10;8, range = 10;2–11;8; 33% females) in Mirandola during March 2014. The earthquake had resulted in damage to persons for 9% of them (injuries to relatives: 3%; relatives’ psychological disturbances: 6%) and economic damage for 71% (to households: 29%; to work activities: 22%; to both: 17%; other kinds: 3%). During 2014, all the children were still attending school in temporary buildings due to structural damage caused by the earthquake.

The control group included 62 children who had not experienced the 2012 Emilia Romagna earthquake and were attending the second grade (*n* = 30; *M*_age_ = 7;9, range = 7;4–8;1; 63% females) or the fifth grade (*n* = 36; *M*_age_ = 10;9, range = 10;3–11;2; 50% females) in a town in the province of Verona during March 2014. This town was similar to Mirandola for characteristics such as population size and geographic region, but it was not hit by either earthquake even though it was situated about 80 km away from its epicenter. None of the children in the control group, therefore, had experienced damage to people or property.

The two groups had similar family characteristics such as fathers’ age (experimental group: *M*_age_ = 45, range = 32–62; control group: *M*_age_ = 43, range = 31–54), mothers’ age (*M*_age_ = 42, range = 30–50; *M*_age_ = 41, range = 30–51, respectively), family composition (92% and 95% of children had two-parent families), and they were from a wide range of socio-economic backgrounds.

### Procedure

The study was carried out following APA ethical guidelines, and it has been approved by the Local Ethical Committee of the Department of Human Science, University of Verona (protocol n. 108200). After authorization had been obtained from each school, we obtained informed, written consent from parents, who also completed a questionnaire on sociodemographic data.

A female researcher interviewed each child in a quiet room of the school, conducting a semi-structured interview about knowledge of earthquakes and associated emotions, adapted from previous works [33,34,47,48]. Concerning knowledge, we asked children to define what an earthquake is (*What is an earthquake*?), and to describe its causes (*What are the causes of an earthquake*?), core happenings (*What happens during an earthquake*?), and consequences (*What happens after the earthquake shocks stop*?). Concerning emotions, we asked children to report people’s emotions associated with them (*When an earthquake occurs*, *how do people feel*?) and to justify their answers (*Why*?). When the children paused, they were prompted with non-directive questions (e.g., *Could you tell me more*?). Finally, we employed a structured task on the intensity of negative emotions associated with earthquakes. See S1 Protocol for the protocol of the semi-structured interview and the structured task in English and in the original language, i.e., Italian. We tape-recorded and transcribed all responses *verbatim*. Before starting data collection, we trialed the tasks with four children (second-graders and fifth-graders) to ensure that the instructions were easily comprehended by children in this age range.

After the interview, a second female researcher tested each child using an instrument measuring the understanding of emotions. Finally, we asked children to deliver to their parents a questionnaire that allowed the parents to report on the extent to which their child (or children) could regulate their emotions. The questionnaires were returned within two weeks from sending them out. At the end of the data collection, each child received a diploma for his/her participation.

### Measures

#### Knowledge of earthquakes

We coded knowledge of earthquakes examining responses on definition, causes, core happenings, and consequences. Knowledge was conceptualized along three dimensions: plausibility, length, and content type, adapting previous schemes [40,48,49]. Specifically:

1. *Plausibility* (0 = *absence* and 1 = *presence*) was defined as the reference to at least one content type pertinent to the concept of earthquake.
2. *Length* was operationalized in terms of number of words [50].
3. *Content types* referred to phenomena, as entities and/or related processes, pertaining to the material domain (natural and man-made aspects) or the individual domain (behavioral, biological, cognitive, and affective aspects), within different temporal frames (distinguishing antecedents, e.g., *It’s the earth that moves*, versus description of the phenomena in terms of consequences, e.g., *Everything is destroyed*). The coding categories were constructed deductively from the literature [33,34,35] and inductively by content analysis of students’ responses [51]. Specifically, content types (0 = *absence* and 1 = *presence*) referred to: (a) *natural* elements, relating to the geological characteristics of earthquakes (e.g., *An earthquake is when the earth vibrates*, *shakes; It’s something that makes the soil tremble*, *which is caused by the lava; Earth’s tectonic plates move and sometimes they collide […] where they collide*, *the terrain begins to tremble*); (b) *man-made* elements, relating to things built by people (e.g., *The houses crack or collapse; Buildings that are not stable can collapse; Furniture*, *school desk*, *the floor move*); (c) *behavioral* elements, relating to people’s behaviors and actions, excluded those related to building things (e.g., *Everybody goes out of home; Or people go to tent camps or they come back home or they go at some relatives’ home; There are volunteers who help people*); (d) *biological* elements, relating to individuals’ physical domains, in terms of reference to their bodies (e.g., *Some people get hurt by a glass falling on them*, *and they cut themselves; Some persons get crushed by the rocks; People could also die*); (e) *cognitive* elements, in terms of thoughts and beliefs (e.g., *I thought “What’s this thing?”; You don’t realize; People try to be more alert*); and (f) *affective* elements, in terms of emotions, feelings, mood, but also references to mental health (e.g., *That everybody feel afraid; You are anxious*, *you are very worried; People can be scared*).

Two judges, blind to the children’s situations including group, class level, and gender, independently coded 30% of the transcripts for reliability. The mean percentage agreement for knowledge of earthquakes was 98% (mean percentage agreement for plausibility: 100%; length: 98%; content types: 95%). Disagreements were solved though discussion between judges. The remaining transcripts were coded by one of them.

#### Emotional language associated with knowledge of earthquakes

Emotional language, as the number of words used in the interview referred to negative and positive emotions [50], was distinguished as (see Table 1 for the complete list):

1. *Negative basic emotions*, including fear (e.g., *afraid*, *scared*, *nervous*), sadness (e.g., *sad*, *depressed*, *unhappy*), and anger (e.g., *angry*, *irritated*, *infuriated*). We included also emotional behaviors, such as *have got the shivers* for fear [50]. Given the absence of spontaneously reported terms on anger, this category was then excluded.
2. *Other negative emotions*, including general state labels (e.g., *to feel bad*, *not to feel well*, *terrible*) and specific terms not included in previous categories (e.g., *unsettled*, *upset*, *discomfort*).
3. *Positive emotions* (e.g., *to feel better*, *calm*, *tranquil*). We did not introduce sub-categories because of their expected low frequency.

**Table 1:** List of emotional language terms (Referred to Fear, Sadness, Other Negative Emotions, Positive Emotions) by question type (General, Specific).

Emotional language	General Questions	Specific Question
Fear	*afraid*, *agitation*, *alarmed*, *anxiety*, *fear*, *scared*, *shock*, *worried*	*afraid*, *agitated*, *agitation*, *anxiety*, *anxious*, *dread*, *fear*, *fearful*, *fright*, *frightened*, *nervous*, *not tranquil*, *panic*, *scare*, *scared*, *shocked*, *tension*, *to cry*, *to fear*, *to have got the shivers*, *worried*, *worry*
Sadness	*desperate*, *sad*	*depression*, *desperate*, *not cheerful*, *not happy*, *sad*, *sadness*, *sorry*, *to cry*, *unhappy*
Other negative emotions	*not beautiful*, *terrible*	*confused*, *dazed*, *discomfort*, *disoriented*, *not beautiful*, *not pleased*, *not to feel good*, *perplexed*, *to feel bad*, *to feel bad inside*, *unsettled*, *upset*, *weird*
Positive Emotions	*calm*, *good*, *to feel better*, *tranquil*	*beautiful*, *happy*, *touched*, *tranquil*

Again, two judges independently coded 30% of the transcripts for reliability. The mean percentage agreement for emotional language was 95% (mean percentage agreement for negative basic emotions: 94%; other negative emotions: 94%; positive emotions: 98%). Disagreements were solved though discussion between judges, and the remaining transcripts were coded by one of them.

#### Emotional intensity

**Fig 1:**
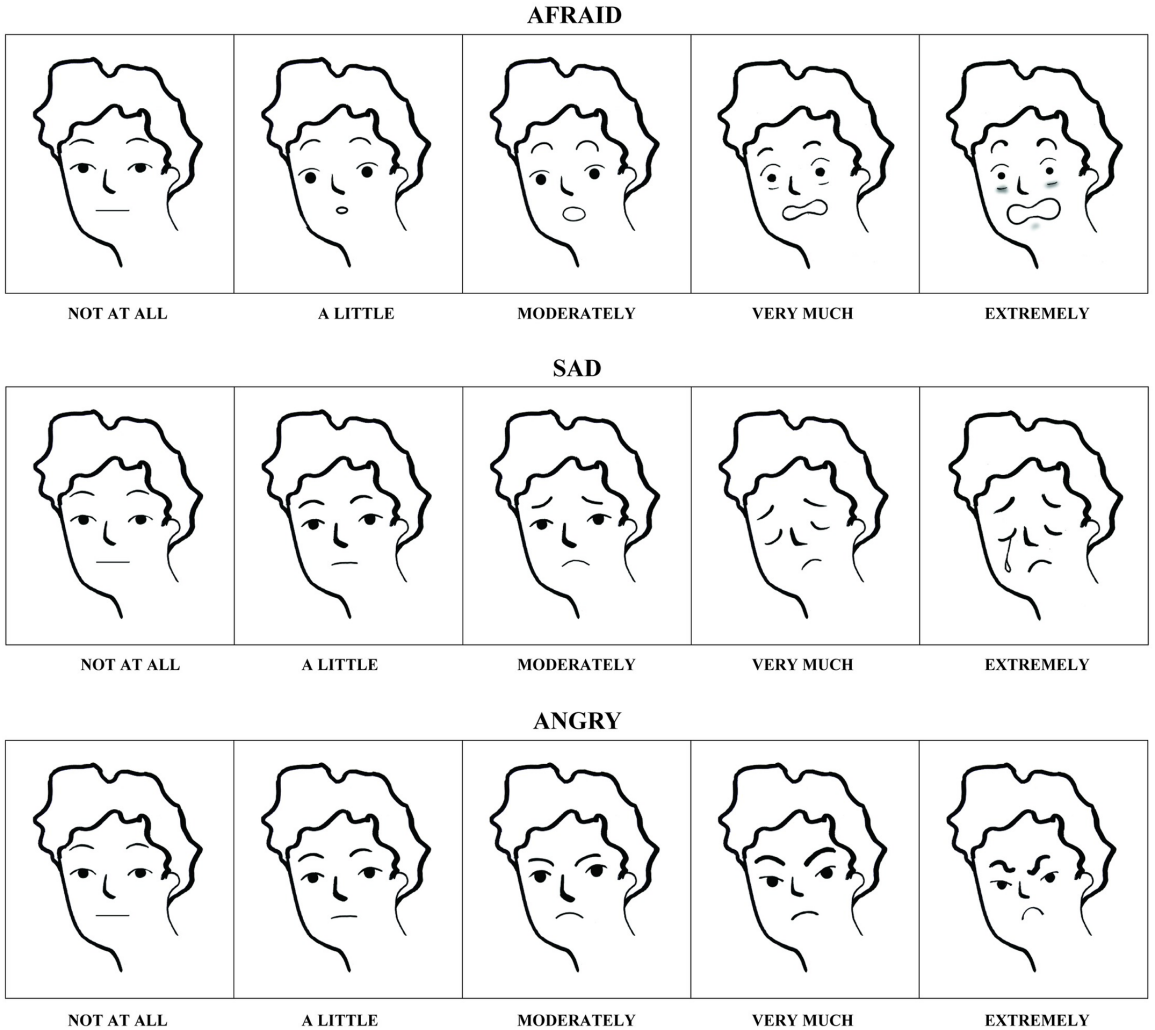
Examples of the 5-point Likert-type scale from the Graduated Achievement Emotions Set (GR-AES) for fear, sadness, and anger, male version.

We used a structured task to assess the intensity of three negative emotions associated with earthquakes, i.e., fear, sadness, and anger, using the Graduated Achievement Emotions Set, GR-AES [52–55]. The GR-AES is a preliminary version of a verbal-pictorial instrument on emotions related to learning, but adaptable to different contexts. For each emotion, children were shown five drawings of facial emotions with increasing intensity (1 = *not at all intense* and 5 = *extremely intense*), and corresponding labels. See Fig 1 for examples of drawings and corresponding labels in English, and Figs A and B in S1 Protocol for all drawings and corresponding labels in English and in the original language (i.e., Italian). The use of a dual-code representation, verbal and pictorial, favors a more direct access to the semantic network in which emotional information is stored [56]. After a familiarization phase, in which all children declared they were acquainted with the presented emotions, each participant indicated the intensity of fear, sadness, and anger a child experiencing an earthquake can feel (i.e., *Think about how a child who is experiencing an earthquake feels*. *According to you*, *how much afraid/sad/angry does s/he feel*?), pointing at the corresponding picture.

#### Understanding of emotions

We evaluated children’s understanding of emotions with the Test of Emotion Comprehension, TEC (see [22] for the Italian version, and [23] for the original language version), developed for three to 12-year-olds children. It assesses understanding referred to nine components: recognition of emotions from facial expressions, understanding of external causes, desire-based and belief-based emotions, influence of reminders, understanding the possibility to regulate and hide emotions, and understanding mixed and moral emotions. For each component, we presented a cartoon scenario asking to choose one among four facial expressions. Each item was evaluated as 0 = *incorrect* or 1 = *correct*. We used forms appropriate to children’s gender [22,25,49].

#### Regulation of emotions

We assessed children’s regulation of emotions by asking their parents to respond using the Emotion Regulation Checklist, ERC (see [25] for the Italian version, and [44] for the original language version), developed for three to 11-year-olds children’ abilities. It is an other-report instrument including 24 items referring to two sub-scales: Emotion Regulation and Lability/Negativity, to be rated on a 4-point Likert-type scale (1 = *almost always* and 4 = *never*). The Emotion Regulation scale refers to the ability to manage one’s emotional arousal to facilitate engagement with the environment, with higher scores corresponding to expression of appropriate emotional reactions, empathy, and self-awareness (eight items; e.g., *S/he is empathic toward others*; *S/he can say when s/he is feeling sad*, *angry or mad*, *fearful or afraid*). The Lability/Negativity scale refers to inappropriate emotional displays, with higher scores indicating exaggerated affective reactions and frequency of mood changes not appropriate to the child’s context (15 items; e.g., *S/he is prone to angry outbursts*; *S/he exhibits wide mood swings*). We used forms appropriate to children’s gender [22,25,49]. We then reversed the Lability/Negativity item scores [44], and we calculated a composite ERC score computing the mean value among all items; for this index Cronbach’s alpha was .75.

### Data analyses

We used Generalized Linear Mixed Models (GLMM). These models provide a flexible approach to analyze dependent variables from different distributions, such as binary, count, or rating data. GLMM are an extension of the set of Generalized Linear Models in which random effects (effects related to individual experimental units randomly selected from a population, that allow to consider variations between groups that might change the dependent variable) are simultaneously considered with fixed factors (the usual linear predictors). This gives the opportunity to analyze clustered data, repeated and longitudinal measurements, multivariate observations, etc., with supple accommodation of covariates. For the aforesaid reasons, we used the lmer/glmer functions in the lme4 package of the R-software environment for statistical computing and graphics [57]. We performed Mixed Model ANOVA Tables (Type 3 tests) via likelihood ratio tests implemented in the afex package of the R-software [58–60]. For significant effects or interactions, we conducted post-hoc tests using the Bonferroni correction, with the lsmeans package [61]. We reported effect sizes in terms of *R*^*2*^ implemented in the MuMIn package [62]. In the case of binary dependent variables, the GLMM used the binomial and logit link-function; for count dependent variables, the GLMM used the Poisson and log link-function; for rating dependent variables, the GLMM used Gaussian and identity link-function. The level of significance was set at *p* < .05. For variables related to the semi-structured interview and the structured task, there were not missing data. For the TEC, there were missing data for one child; for the ERC, there were missing data for ten children. We conducted the corresponding analyses with listwise exclusion. See S1 Dataset for consulting the whole data.

For the experimental group, preliminary analyses revealed no differences related to the type of damage (to persons, economic).

## Results

### Understanding of emotions and their regulation: Group, class level, and gender differences

#### Understanding of emotions

We considered group (experimental, control), class level (second, fifth-graders), and gender (males, females) as fixed effects; participants as the random effect; and TEC item scores as binary dependent variables (*R*^*2*^ = .44). The model did not yield a significant effect of group.

However, we found significant effects of class, *Χ*^*2*^(1) = 26.34, *p* < .001, and gender *Χ*^*2*^(1) = 4.99, *p* = .002. The score was lower for second-graders (*M* = 0.83, *SE* = 0.51, 95% CI: 0.64 to 0.93) compared to fifth-graders (*M* = 0.93, *SE* = 0.52, 95% CI: 0.84 to 0.97), and for males (*M* = 0.87, *SE* = 0.51, 95% CI: 0.71 to 0.95) compared to females (*M* = 0.91, *SE* = 0.52, 95% CI: 0.79 to 0.97). In addition, a significant group X class X gender interaction, *Χ*^*2*^(1) = 5.99, *p* = .014, indicated that class differences were particularly marked for females in the experimental group, *z* = -3.32, *p* = .024 (second-graders: *M* = 0.82, *SE* = 0.54, 95% CI: 0.61 to 0.93; fifth-graders: *M* = 0.95, *SE* = 0.62, 95% CI: 0.85 to 0.98), and for males in the control group, *z* = -4.19, *p* < .001 (*M* = 0.73, *SE* = 0.56, 95% CI: 0.48 to 0.89; *M* = 0.94, *SE* = 0.58, 95% CI: 0.83 to 0.98, respectively).

#### Regulation of emotions

We included group, class, and gender as fixed effects; participants as the random effect; and ERC item scores as rating dependent variables (*R*^*2*^ = .34). The analysis revealed no significant differences in regulation of emotions between the experimental group (*M* = 3.34, *SE* = 0.10, 95% CI: 3.14 to 3.53) and the control group (*M* = 3.32, *SE* = 0.10, 95% CI: 3.13 to 3.52).

No significant effects emerged for the other factors.

### Knowledge of earthquakes: Group, class level, and gender differences

#### Plausibility of knowledge of earthquakes as coded from the semi-structured interview

We checked for differences in plausibility (as binary dependent variable) studying group, class, and gender as fixed effects, and participants as the random effect (*R*^*2*^ = .96). The plausibility of children’s knowledge did not differ according to their exposure to the earthquake.

No other significant effect emerged. Most of the participants (experimental group: *M* = 0.98, *SE* = 1.01, 95% CI: 0.90 to 1.00; control group: *M* = 0.97, *SE* = 1.72, 95% CI: 0.88 to 0.99) referred to at least one content type pertinent to the concept of earthquake.

#### Length of the semi-structured interview on knowledge of earthquakes

Again, we controlled for differences in the length (as count dependent variable) of children’s responses considering group, class, and gender as fixed effects, and participants as the random effect (*R*^*2*^ = .11). Significant differences between groups emerged, *Χ*^*2*^(1) = 4.40, *p* = .035: Length was higher for the experimental group (*M* = 59.52, *SE* = 8.53, 95% CI: 42.64 to 76.40) compared to the control group (*M* = 35.59, *SE* = 8.45, 95% CI: 18.86 to 52.32).

Also class level, *Χ*^*2*^(1) = 5.95, *p* = .014, resulted significant: Older children (*M* = 61.46, *SE* = 8.23, 95% CI: 45.16 to 77.76) gave longer answers compared to younger children (*M* = 32.14, *SE* = 8.78, 95% CI: 14.76 to 49.52).

#### Content types of the semi-structured interview on knowledge of earthquakes

**Fig 2:**
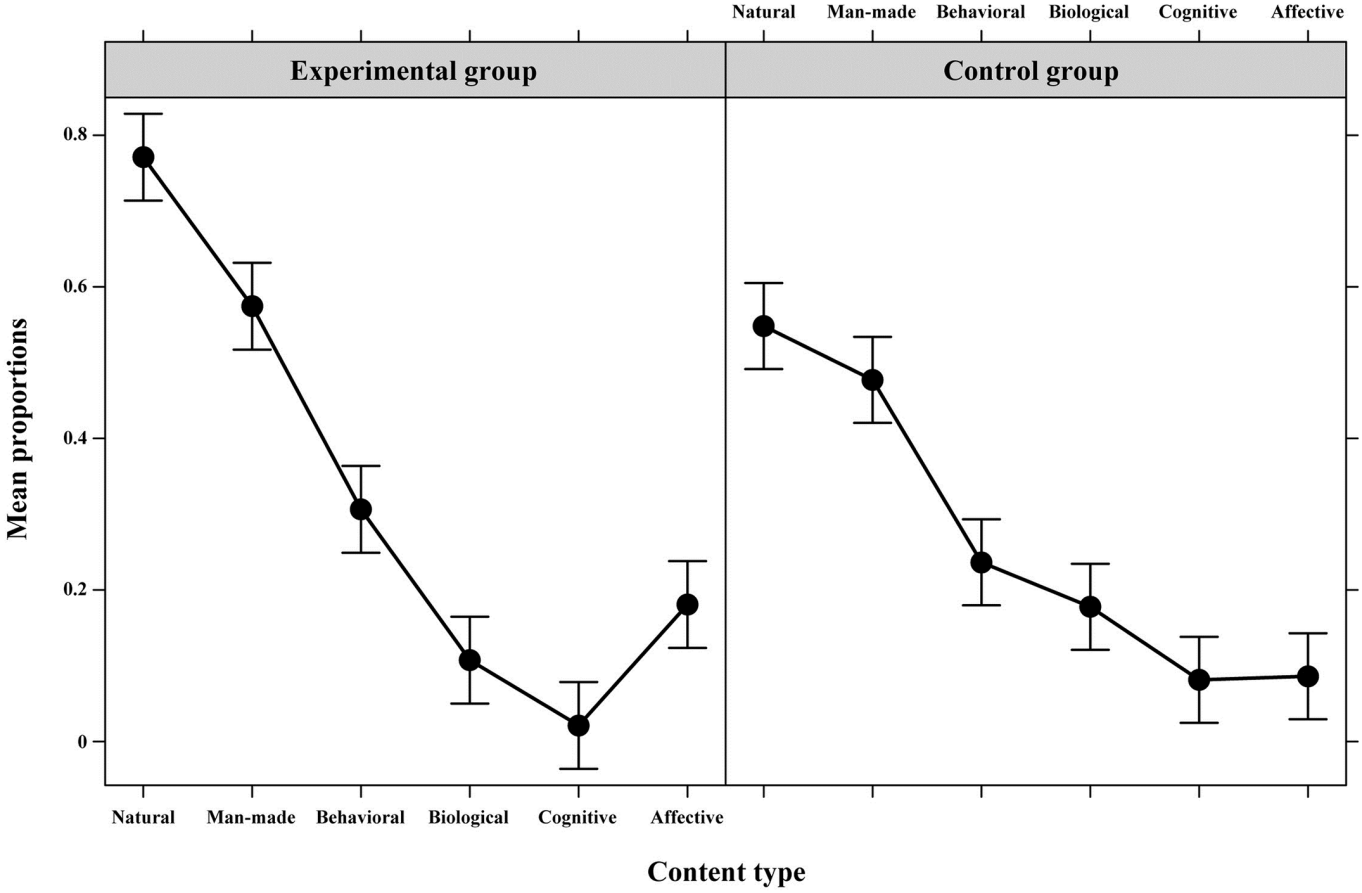
Mean proportions (95% Confidence Interval) of different content types (Natural, Man-made, Behavioral, Biological, Cognitive, Affective), focused on antecedents or consequences, in children’s responses, separately by group (Experimental, Control).

We considered group, class, gender, content type (natural, man-made, behavioral, biological, cognitive, affective), and temporal frame (antecedents, consequences) as fixed effects; participants as the random effect; and presence of each content type as binary dependent variables (*R*^*2*^ = .97). We found a significant effect of group, *Χ*^*2*^(1) = 3.81, *p* = .049: The presence of content types focused on antecedents or consequences was higher for the experimental (*M* = 0.32, *SE* = 0.76, 95% CI: 0.29 to 0.36) compared to the control group (*M* = 0.27, *SE* = 0.82, 95% CI: 0.24 to 0.30). Also, a significant effect of content type, *Χ*^*2*^(5) = 130.70, *p* < .001, emerged, moderated by a significant group X content type interaction, *Χ*^*2*^(5) = 17.82, *p* = .003 (see Table 2 for *z*-scores; see Fig 2 for mean proportions). On the one hand, post-hoc tests indicated that for both groups material aspects were more frequent than individual aspects. However, in the experimental group children revealed a more differentiated conceptualization of earthquakes. They also referred more frequently to natural aspects (*M* = 0.77, *SE* = 0.21, 95% CI: 0.69 to 0.84) versus man-made aspects (*M* = 0.57, *SE* = 0.18, 95% CI: 0.48 to 0.65); to behavioral aspects (*M* = 0.31, *SE* = 0.19, 95% CI: 0.23 to 0.39) versus biological (*M* = 0.11, *SE* = 0.29, 95% CI: 0.06 to 0.17) and cognitive aspects (*M* = 0.02, *SE* = 0.58, 95% CI: 0.01 to 0.07); and to affective aspects (*M* = 0.17, *SE* = 0.23, 95% CI: 0.12 to 0.24) versus cognitive aspects. Such differences did not emerge for the control group (natural: *M* = 0.56, *SE* = 0.18, 95% CI: 0.47 to 0.64; man-made: *M* = 0.48, *SE* = 0.18, 95% CI: 0.39 to 0.56; behavioral: *M* = 0.23, *SE* = 0.22, 95% CI: 0.17 to 0.32; biological: *M* = 0.18, *SE* = 0.23, 95% CI: 0.12 to 0.26; affective: *M* = 0.09, *SE* = 0.32, 95% CI: 0.05 to 0.15; cognitive: *M* = 0.08, *SE* = 0.33, 95% CI: 0.04 to 0.14). On the other hand, post-hoc tests confirmed differences between groups for natural contents, more frequent for the experimental group, *z* = 3.52, *p* = .028.

**Table 2:** *Z*-scores and *p*-values concerning post-hoc tests comparing content types, separately by group (Experimental, Control).

Comparisons between Content Types	Experimental Group		Control Group	
	*z*	*p*	*z*	*p*
Natural—Man-Made	3.40	.045	1.28	1.000
Natural—Behavioral	7.13	< .001	5.08	< .001
Natural—Biological	9.41	< .001	5.82	< .001
Natural—Affective	8.88	< .001	7.03	< .001
Natural—Cognitive	7.99	< .001	7.08	< .001
Man-Made—Behavioral	4.20	.002	3.93	.006
Man-Made—Biological	7.17	< .001	4.74	< .001
Man-Made—Affective	6.37	< .001	6.15	< .001
Man-Made—Cognitive	6.61	< .001	6.23	< .001
Behavioral—Biological	3.83	.008	0.94	1.000
Behavioral—Affective	2.60	.619	3.01	.171
Behavioral—Cognitive	4.79	< .001	3.19	.094
Biological—Affective	-1.43	1.000	2.18	1.000
Biological—Cognitive	2.52	.782	2.37	1.000
Affective—Cognitive	3.43	.039	0.23	1.000

In addition, we found significant effects for class, *Χ*^*2*^(1) = 10.80, *p* = .001, and temporal frame, *Χ*^*2*^(1) = 113.42, *p* < .001. The percentage of content types was lower for second-graders (*M* = 0.25, *SE* = 0.09, 95% CI: 0.22 to 0.28) compared to fifth-graders (*M* = 0.34, *SE* = 0.07, 95% CI: 0.31 to 0.37), and for those focused on antecedents (*M* = 0.10, *SE* = 0.12, 95% CI: 0.08 to 0.12) rather than consequences (*M* = 0.49, *SE* = 0.07, 95% CI: 0.46 to 0.53).

### Emotions associated with earthquakes

#### Emotional language associated with knowledge of earthquakes

Group, class, gender, type of emotional language (fear, sadness, other negative emotions, positive emotions), and question type (first four general questions, question focused on emotions) were the fixed effects; participants the random effect; and presence of each type of emotional terms the count dependent variables (*R*^*2*^ = .92). First, significant effects of group, *Χ*^*2*^(1) = 3.91, *p* = .048, and class, *Χ*^*2*^(1) = 7.70, *p* = .005, emerged, moderated by a group X class interaction, *Χ*^*2*^(1) = 6.69, *p* = .009. Overall, emotional terms were more frequent for the experimental (*M* = 0.36, *SE* = 0.09, 95% CI: 0.30 to 0.43) compared to the control group (*M* = 0.26, *SE* = 0.10, 95% CI: 0.21 to 0.31), and for older (*M* = 0.24, *SE* = 0.10, 95% CI: 0.19 to 0.29) compared to younger children (*M* = 0.38, *SE* = 0.08, 95% CI: 0.32 to 0.44). However, post-hoc tests indicated that group differences characterized fifth (experimental: *M* = 0.48, *SE* = 0.10, 95% CI: 0.39 to 0.58; control: *M* = 0.28, *SE* = 0.13, 95% CI: 0.22 to 0.36; *z* = 3.38, *p* = .004) but not second-graders (experimental: *M* = 0.24, *SE* = 0.14, 95% CI: 0.18 to 0.32; control: *M* = 0.25, *SE* = 0.14, 95% CI: 0.19 to 0.32).

Then, the analysis revealed that emotional terms differed according to their type, *Χ*^*2*^(3) = 174.64, *p* < .001. They were more frequent for fear (*M* = 0.95, *SE* = 0.07, 95% CI: 0.82 to 1.10) compared to sadness (*M* = 0.08, *SE* = 0.21, 95% CI: 0.06 to 0.13; *z* = 11.21, *p* < .001), other negative emotions (*M* = 0.15, *SE* = 0.16, 95% CI: 0.11 to 0.21; *z* = 11.04, *p* < .001), and positive emotions (*M* = 0.04, *SE* = 0.29, 95% CI: 0.02 to 0.08; *z* = 10.48, *p* < .001). Also, other negative emotion terms were more frequent than positive emotion terms, *z* = 3.77, *p* = .001. Finally, emotional terms, *Χ*^*2*^(1) = 61.07, *p* < .001, were more frequent in response to questions focused specifically on emotions (*M* = 0.50, *SE* = 0.07, 95% CI: 0.43 to 0.58) rather than general knowledge of earthquake (*M* = 0.12, *SE* = 0.13, 95% CI: 0.09 to 0.15). However, as suggested by a significant question type X class interaction, *Χ*^*2*^(1) = 4.37, *p* = .036, class differences were revealed for general questions (second-graders: *M* = 0.04, *SE* = 0.33, 95% CI: 0.02 to 0.07; fifth-graders: *M* = 0.19, *SE* = 0.14, 95% CI: 0.14 to 0.25; *z* = -4.53, *p <* .001) but not for specific questions (second-graders: *M* = 0.44, *SE* = 0.11, 95% CI: 0.36 to 0.55; fifth-graders: *M* = 0.56, *SE* = 0.09, 95% CI: 0.47 to 0.67).

#### Emotional intensity of negative emotions associated with earthquakes

We explored differences in emotional intensity (as rating dependent variable) considering group, class, gender, and emotion (fear, sadness, anger) as fixed effects, and participants as the random effect (*R*^*2*^ = .55). The model revealed differences related to group, *Χ*^*2*^(1) = 7.31, *p* = .006: Intensity was higher for the experimental (*M* = 3.56, *SE* = 0.11, 95% CI: 3.35 to 3.78) compared to the control group (*M* = 3.18, *SE* = 0.11, 95% CI: 2.96 to 3.40).

In addition, emotional intensity, *Χ*^*2*^(2) = 313.43, *p* < .001, was stronger for fear (*M* = 4.43, *SE* = 0.11, 95% CI: 4.22 to 4.64; *z* = 6.26, *p* < .001) compared to sadness (*M* = 3.57, *SE* = 0.11, 95% CI: 3.36 to 3.79), and for sadness compared to anger (*M* = 2.12, *SE* = 0.11, 95% CI: 1.91 to 2.33; *z* = 10.38, *p* < .001). It also depended on class, *Χ*^*2*^(1) = 11.85, *p* < .001, being it higher for second (*M* = 3.62, *SE* = 0.12, 95% CI: 3.39 to 3.84) compared to fifth-graders (*M* = 3.17, *SE* = 0.11, 95% CI: 2.96 to 3.38). However, a significant class X emotion interaction, *Χ*^*2*^(2) = 12.40, *p* = .002, suggested that class differences characterized only anger (second-graders: *M* = 2.64, *SE* = 0.15, 95% CI: 2.34 to 2.95; fifth-graders: *M* = 1.66, *SE* = 0.14, 95% CI: 1.38 to 1.94; *z* = 4.70, *p* < .001).

## Discussion

This study focused on the long-term impact of experiencing the 2012 Emilia Romagna earthquake on children’s emotional competence, in terms of understanding, regulating, and expressing emotions [10]. We compared these children with a group of children who had not been exposed to the same disaster, acknowledging methodological difficulties in establishing comparable groups [9]. Much is known about post-disaster reactions in terms of children’s mental health [4,6–9,11,13–16], but empirical evidence about how these might change with development is limited [9], especially focusing on the emotional domain (for exceptions, see [21,33]). Gathering empirical data on this issue could also help to extend the generalizability of existing theoretical models of children’s psychological functioning following disasters, such as the dose-response model [6,11,14] and the organizational-developmental model for child resilience [21]. At an applied level, documenting how exposure affects children’s emotional development enables both professionals and volunteers to be aware of children’s resources as a preliminary step to helping them to cope with traumatic events.

We found that the understanding of emotions and their regulation did not vary with exposure to the earthquake; a finding that differs from those associated with maltreated children [24,26]. This result suggests that experiencing the 2012 Emilia Romagna earthquake seems not to have impaired children’s emotional development in terms of these two abilities after two years. It is worth noting that only a small percentage of the children involved had experienced damage to persons (i.e., injuries or psychological disturbances for relatives), while a greater percentage had experienced some kind of economic damage. Differences from findings on maltreated children could be linked to the fact that those children are forced to reframe their psychological representation of the emotional bonds with other people, frequently significant ones, who are responsible for their traumas, while this is not the case when a traumatic event is due to natural phenomena, even when some responsibility for damage could be attributed to humans. In line with documented developmental trends [22,23], understanding of emotions was better for older children; these differences were particularly marked for girls who experienced the earthquake and boys who did not experience it. In addition, our data revealed further gender effects, for which the literature frequently reported inconsistent results. Girls outperformed boys on understanding measures, an effect not expected on the bases of previous TEC studies [22,23] but not surprising when considering findings, for example, on females’ richer abilities to use emotional terms [42]. As we had anticipated, no class level or gender effects emerged for regulation. This could be due to an anchoring effect where parents evaluated the adequacy of their children’s strategies against a perceived model typical of a specific developmental phase or gender [25,26].

When asked about earthquakes using open-ended prompts, most of the children demonstrated familiarity with the concept, reporting at least one plausible content, and length and complexity of content types were higher at increasing ages. This supported our hypotheses based on development related to both cognitive, linguistic, and emotional abilities, and specific knowledge [9,33–35,45,46]. We also found that antecedents of earthquakes were less frequently cited than the immediate manifestation and consequences. At an applied level, this supports the need for education designed to enhance children’s scientific knowledge about the causal mechanisms underlying natural phenomena. This is particularly relevant, given the documented links between knowledge and propensity to act to prevent earthquakes damage, at least for adults [28,29].

Our findings indicated that exposure to an earthquake markedly influenced the complexity of children’s representation of such an event: For both groups, children referred more frequently to material rather than individual aspects, but for the experimental group the conceptualization was much more differentiated. On the one hand, all of the children frequently reported geological elements, demonstrating their basic understanding of the phenomenon at issue. Man-made elements were also salient (even if less salient than geological elements for the experimental group), indicating children’s realistic grasp of one of the first-level negative consequences of an earthquake–the damage or destruction of infrastructure such as roads and buildings. This suggests a preliminary understanding of the concept of risk, not directly related to the occurrence of a natural phenomenon such as the earth shaking, but rather to the presence of people and human-made structures in the area in which it occurs.

In the experimental group children differentiated individual elements in a more complex way than in the control group. They referred more often to actions that people can carry out than to individuals’ biological and cognitive domains. There were few references to death and injuries, which could be linked to children’s rare direct experience of these events. Nevertheless, references to affective elements indicates that, while these kinds of elements are not central in the structure of scripts [30,31], they play a role in the representation of earthquakes. It may be that the events experienced by children in the experimental group led them to rely more on behaviors rather than facts when constructing their scripts [32], with subsequent increase in script complexity [30]. We suggest that further studies should explore the mechanisms responsible for the links between experiencing an earthquake and the enrichment of the related semantic knowledge. For example, in line with what was observed in the study of autobiographical memories of traumatic events such as the hurricane Andrew, our findings could be explained by the inverted “U” relation between stress and recall [63]. In our case, we might consider the control and experimental groups as having experienced low and moderate levels of stress respectively [31]. However, more data are needed to generalize our findings. In addition, it is worth noting that in this work we could not have a complete picture of the factors that contributed to children’s differences in earthquake related knowledge. For example, differences may indeed reflect the degree to which important adults such as parents, caregivers, or teachers discussed the events, explained what happened, discussed the children’s reactions and responses, shared their own responses, etc. Parents and other adults could also help promote specific coaching approaches, provide warmth and support in the aftermath of the event, and assume other important functions, which could have played a role in elaborating emotional information.

Acknowledging these limitations, we found that the level of exposure to earthquakes was a key factor in explaining differences in children’s emotions associated with earthquakes, as revealed by analyzing emotional language and intensity. Children belonging to the experimental group spontaneously reported a higher number of emotions but this effect emerged only for older children, and they expressed fear, sadness, and anger more intensely, than did the control group. Our findings extend previous literature which has shown that children’s long-term memory of traumatic events, such as tornados, is richer in terms of negative emotions when the event is experienced directly [38].

Different interpretations could be proposed to account for these data. It is probable that children in the experimental group really experienced the negative emotions they cited, and therefore our results could mirror differences in encoded information and/or long-term negative consequences of having experienced a traumatic event as has been documented for mental health disturbances [6–9,11]. Data on the presence of PTSD among the involved children (not gathered for this work) could have helped to understand whether differences in children’s emotions reflect a richer and more realistic elaboration of the traumatic event, or rather are indicators of negative emotional symptoms, as has been seen when projective techniques are used [13]. However, the absence of differences in understanding and regulation of emotions between the two groups partially supports the first interpretation. In other words, it could be that children exposed to the earthquake had a more realistic representation of such a traumatic event and were more aware, as happen with adults, of the salience of negative emotions in such events without being necessarily impaired in their emotional functioning. Further studies should be conducted to disambiguate this issue.

Finally, it is worth noting that all of the children were able to master the emotions necessary to describe events that can have an affective impact on people; an ability that increased with age as has been previously reported [36,37,45,46]. As expected, fear was reported as the most frequent and evaluated as the most intense emotion, followed by sadness and anger. These findings are consonant with those in the general trauma literature which reports the centrality of emotions signifying fear, such as anxiety, in both post disaster symptomatology and normal reactions [6,7,17]. It is also worth noting that anger was evaluated as particularly intense by younger compared to older children, suggesting that the kind of post-disaster symptoms specific for an age level (i.e., aggressive reactions are more typical during preschool years [13]) could play a role in the delayed representation of the traumatic event.

Despite the theoretical relevance of our findings, this study suffers from several limitations requiring further attention in future research. Given the exploratory nature of the study, the nature and size of the convenience sample limited the scope of the possible analyses (as in [13]). In addition, possible null or small effects could be due to the small sample size. No records were available to allow us to gauge the mental health of the children both prior to or after the earthquake; a limitation that is quite common in research focused on unanticipated disasters [9]. The study design was cross-sectional, and there were practical limits to the extent that we could equate the experimental and control groups. In addition, collecting our data two years after the earthquake could be problematic for the interpretation of our results, given that the lack of significant differences between groups on some of the variables may be due to the passage of time–in many cases of child exposure to disaster and war, symptoms diminish over time. A longitudinal research design with the same variables measured both just after the earthquake and after two years could have helped to obtain more valid findings on the consequences of earthquakes for children, but practical limitations made it not possible. Finally, our measures were limited by constraints typical of dichotomous or ordinal observations [64]. However, most of these limitations frequently characterize reported research on the impact of disasters on children, making research in this field particularly challenging [9].

## Conclusion

Our findings indicate that exposure to the 2012 Emilia Romagna earthquake enriched children’s representation of earthquakes, both in terms of knowledge and associated emotions, and it seemed not to have impaired their understanding and regulation of emotions after a long-term time interval. They extend the generalizability of theoretical models on children’s psychological functioning following disasters, such as the dose-response model [6,11,14] and the organizational-developmental model for child resilience [21]. Beyond their theoretical relevance, these results may further inform the planning of efficacious education programs, in which awareness of children’s emotional resources plays a fundamental role.

## Supporting information

S1 Protocol Protocols of semi-structured interview and structured task. Protocols of the semi-structured interview about knowledge of earthquakes and associated emotions, and the structured task on the intensity of negative emotions associated with earthquakes. English and original language (i.e., Italian) versions.
(DOCX)

S1 Dataset S1 Dataset of the study. (XLSX)
