# Supplementary material for: Children's emotional experience two years after an earthquake: An exploration of knowledge of earthquakes and associated emotions
Source: PLoS One. 2017 Dec 20;12(12):e0189633. doi: 10.1371/journal.pone.0189633 (PMC5738038; doi:10.1371/journal.pone.0189633)
Supplement: S1 Protocol — Protocols of the semi-structured interview about knowledge of earthquakes and associated emotions, and the structured task on the intensity of negative emotions associated with earthquakes. English and original language (i.e., Italian) versions. (DOCX) [file pone.0189633.s001.docx]

**Protocols of Semi-structured Interview and Structured Task**

**Semi-structured Interview about Knowledge of Earthquakes and Associated Emotions**

**English Version**

*This research relates to how people, and in particular children, think about some things. Now I will ask you some questions. For me it is important that you think about the answers before replying. There are not right or wrong answers, each child answers as s/he prefers. If you don’t know immediately how to answer, please think about it. If you don’t have anything to say, please tell me. If you have any question, please tell me.*

1. *What is an earthquake? Could you tell me more?*

2. *What are the causes of an earthquake? Could you tell me more?*

3. *What happens during an earthquake? Could you tell me more?*

4. *What happens after the earthquake shocks stop? Could you tell me more?*

5. *When an earthquake occurs, how do people feel? Could you tell me more? And why do they feel so?* *Could you tell me more?*

**Original Language (i.e., Italian) Version**

*Questa ricerca si occupa di come la pensano le persone, e in particolare i bambini, su alcune cose. Adesso io ti faccio alcune domande. Per me è importante che ci pensi bene prima di rispondere. Non ci sono risposte giuste o sbagliate, ogni bambino risponde come vuole. Se non ti viene in mente subito cosa dire, pensaci pure. Se proprio non ti viene in mente altro, dimmelo. Se ti viene in mente qualche domanda da farmi, fammela pure.*

1. *Che cos’è un terremoto? Ti viene in mente qualcos’altro?*

2. *Quali sono le cause dei terremoti? Ti viene in mente qualcos’altro?*

3. *Che cosa succede durante un terremoto? Ti viene in mente qualcos’altro?*

4. *Che cosa succede dopo che ci sono state le scosse di un terremoto? Ti viene in mente qualcos’altro?*

5. *Durante un terremoto, come si sentono le persone che si trovano lì? Ti viene in mente qualcos’altro? E come mai si sentono così? Ti viene in mente qualcos’altro?*

**Structured Task on the Intensity of Negative Emotions associated with Earthquakes**

**English Version**

*Now I will show you some faces that represent children who feel different emotions (Fig. A). Please tell me if there are emotions that you don’t know. This an afraid face. This is a sad face. This is an angry face. Ok? This child is not afraid at all, this child is a little afraid, this child is moderately afraid, this child is very much afraid, this child is extremely afraid. This child is not sad at all, this child is a little sad, this child is moderately sad, this child is very much sad, this child is extremely sad. This child is not angry at all, this child is a little angry, this child is moderately angry, this child is very much angry, this child is extremely angry. Ok?*

*Now think about how a child who is experiencing an earthquake feels.*

*6. According to you, how much afraid does s/he feel? Please point at the face.*

*7. According to you, how much sad does s/he feel? Please point at the face.*

*8. According to you, how much angry does s/he feel? Please point at the face.*

**Original Language (i.e., Italian) Version**

*Adesso ti mostro delle facce che rappresentano dei bambini che provano diverse emozioni (Fig. B). Dimmi pure se c’è qualche emozione che non conosci. Questa è una faccia spaventata. Questa è una faccia triste. Questa è una faccia arrabbiata. Ok? Questo/a bambino/a non è per niente spaventato/a, questo/a bambino/a è un po’ spaventato/a, questo/a bambino/a è abbastanza spaventato/a, questo/a bambino/a è molto spaventato/a, questo/a bambino/a è spaventato/a moltissimo. Questo/a bambino/a non è per niente triste, questo/a bambino/a è un po’ triste, questo/a bambino/a è abbastanza triste, questo/a bambino/a è molto triste, questo/a bambino/a è triste moltissimo. Questo/a bambino/a non è per niente arrabbiato/a, questo/a bambino/a è un po’ arrabbiato/a, questo/a bambino/a è abbastanza arrabbiato/a, questo/a bambino/a è molto arrabbiato/a, questo/a bambino/a è arrabbiato/a moltissimo. Ok?*

*Adesso pensa a come si sente un/a bambino/a durante un terremoto.*

*6. Secondo te, quanto spaventato/a si sente? Indicamelo con un dito.*

*7. Secondo te, quanto triste si sente? Indicamelo con un dito.*

*8. Secondo te, quanto arrabbiato/a si sente? Indicamelo con un dito.*

**Fig A***.* **Five-point Likert-type Scale from the Graduated Achievement Emotions Set (GR-AES) for Fear, Sadness, and Anger, Male and Female Version (English version).**

**Fig B***.* **Five-point Likert-type Scale from the Graduated Achievement Emotions Set (GR-AES) for Fear, Sadness, and Anger, Male and Female Version (Italian version)**
